# Role of miR-24 in Multiple Endocrine Neoplasia Type 1: A Potential Target for Molecular Therapy

**DOI:** 10.3390/ijms22147352

**Published:** 2021-07-08

**Authors:** Francesca Marini, Maria Luisa Brandi

**Affiliations:** 1 Department of Experimental and Clinical Biomedical Sciences, University of Florence, Viale Pieraccini 6, 50139 Florence, Italy; francesca.marini@unifi.it; 2 F.I.R.M.O., Italian Foundation for the Research on Bone Diseases, Via Reginaldo Giuliani 195/A, 50141 Florence, Italy

**Keywords:** multiple endocrine neoplasia type 1 (MEN1), *MEN1* gene, loss of heterozygosity (LOH), microRNA (miRNAs), miR-24

## Abstract

Multiple endocrine neoplasia type 1 (MEN1) is a rare autosomal dominant inherited multiple cancer syndrome of neuroendocrine tissues. Tumors are caused by an inherited germinal heterozygote inactivating mutation of the *MEN1* tumor suppressor gene, followed by a somatic loss of heterozygosity (LOH) of the *MEN1* gene in target neuroendocrine cells, mainly at parathyroids, pancreas islets, and anterior pituitary. Over 1500 different germline and somatic mutations of the *MEN1* gene have been identified, but the syndrome is completely missing a direct genotype-phenotype correlation, thus supporting the hypothesis that exogenous and endogenous factors, other than *MEN1* specific mutation, are involved in MEN1 tumorigenesis and definition of individual clinical phenotype. Epigenetic factors, such as microRNAs (miRNAs), are strongly suspected to have a role in MEN1 tumor initiation and development. Recently, a direct autoregulatory network between miR-24, *MEN1* mRNA, and menin was demonstrated in parathyroids and endocrine pancreas, showing a miR-24-induced silencing of menin expression that could have a key role in initiation of tumors in MEN1-target neuroendocrine cells. Here, we review the current knowledge on the post-transcriptional regulation of *MEN1* and menin expression by miR-24, and its possible direct role in MEN1 syndrome, describing the possibility and the potential approaches to target and silence this miRNA, to permit the correct expression of the wild type menin, and thereby prevent the development of cancers in the target tissues.

## 1. Introduction

Multiple endocrine neoplasia type 1 (MEN1) is a rare autosomal dominant inherited cancer syndrome that causes the development of multiple endocrine and non-endocrine tumors in a single patient [1,2]. The main affected organs are parathyroid glands, anterior pituitary, and the neuroendocrine cells of the gastro-entero-pancreatic tract. Morbidity and mortality of the disease are related to hormone over-secretion by endocrine functioning tumors, leading to the development of specific syndromes, and/or to the malignant progression of silent tumors, such as non-functioning neuroendocrine neoplasms of the pancreas and the thymus.

Medical therapies of MEN1 aim to control hormone over-secretion and tumor growth. Surgery is the main treatment employed for parathyroid adenomas and gastro-entero-pancreatic neuroendocrine tumors (GEP-NETs) [3]. No therapeutic intervention is definitively resolutive; given the genetic nature of the syndrome and the asynchronous development of tumors, MEN1 patients have a high prevalence of post-operative tumor recurrences, both in the parathyroids and the gastro-entero-pancreatic tract [4]. Therefore, there is a strong need for novel therapies acting at the molecular level and able to prevent tumors in the target neuroendocrine cells. The comprehension of molecular mechanisms underlying MEN1 tumorigenesis is fundamental to identify possible targets for the design of novel therapies [2].

In 1997, the causative gene, *MEN1*, was identified at the 11q13 locus [5]. The *MEN1* gene is a classic tumor suppressor gene: The first inactivating heterozygote mutation is inherited by the affected parent (first hit), while the second copy of the gene is somatically lost in target neuroendocrine cells (second hit), mainly by a large deletion at the 11q13 locus or, more rarely, by a second intragenic loss-of-function mutation (loss of heterozygosity; LOH) [6,7]. The *MEN1* gene encodes menin, a nuclear protein which exerts a wide spectrum of key activities, such as control of cell cycle and apoptosis, regulation of gene transcription and chromatin structure, and DNA repair [8]. Loss of both wild type *MEN1* copies, resulting in loss of menin functions, appears to be the trigger of tumor initiation in MEN1 target neuroendocrine cells. However, the absence of a complete genotype-phenotype correlation and the different tumor manifestations between carriers of the same *MEN1* mutation (even homozygote twins) suggest that other factors concur to cause MEN1 individual tumorigenesis. Epigenetic factors are the main suspected co-actors in driving tumor development and progression in MEN1 target neuroendocrine cells [9]. Alterations in the normal epigenetic regulation of gene transcription (histone modification and/or DNA methylation), following the loss of wild type menin activity, have been demonstrated to play an important role in the progression of MEN1 pancreatic neuroendocrine tumors [10].

Among epigenetic regulators of gene expression, microRNAs (miRNAs) have recently been shown to be involved in the development of various human malignancies, either acting directly as oncogenes (oncomiRs) or inhibiting the expression of tumor suppressor genes [11]. These molecules are non-coding small RNAs that normally negatively regulate gene expression by directly binding the 3′UTR of their target mRNAs [12,13,14]. Through the activity of tissue- and cell-specific miRNAs, the organism regulates the expression of numerous genes, in a spatial and temporal way, granting the correct functionality of various and important biological processes [15,16]. Alterations of expression and/or activity of one or more miRNAs can lead to disease development, including cancer. A role of miRNAs has been demonstrated in the initiation of various human malignancies [17,18,19] and in development of metastases [20,21].

In the last two decades, tissue-specific altered activity and/or expression of miRNAs have been suggested as possible modulators of MEN1 tumorigenesis [22,23,24,25], acting synergically with the *MEN1* mutation, indicating the miR-24 as a possible effector of tumor development.

Here, we review results from recent studies that demonstrate the existence of an autoregulatory network between miR-24, *MEN1* mRNA, and menin, suggesting possible roles of this miRNA in MEN1 tumorigenesis, and we discuss the possibility to silence this molecule in *MEN1* mutation carriers to prevent/reduce cancer development and/or progression.

## 2. The Autoregulatory Network between miR-24, *MEN1*, and Menin: A Possible Effector of MEN1 Tumorigenesis

miR-24 is encoded by two separated chromosomal locations: one gene cluster located on chromosome 9q22, which includes miR-23b, miR-27b, and miR-24-1; and a second gene cluster located on chromosome 19p13, which includes miR-23a, miR-27a, and miR-24-2 [26]. The two miR-24 mature isoforms are identical in nucleotide sequencing, regardless of the different chromosomal origin.

Using Miranda, Target Scan, and Pictar Vert prediction software, Luzi et al. predicted the 599-605 nt position of the 832 nt-3′UTR of *MEN1* mRNA as a direct target of miR-24-1 [27]. The seed site of miR-24-1, which binds to *MEN1* mRNA 3′UTR, is highly conserved in humans, rats, mice, chickens, and dogs. The direct targeting of *MEN1* mRNA was demonstrated in vitro for the first time in 2012 by Luzi et al. [27] in the BON1 cells, a human cell line derived from a lymph node metastasis of a serotonin-secreting pancreatic neuroendocrine tumor [28], via luciferase report assays. Authors showed that the induced over-expression of miR-24-1, through the transfection of pri-miR-24, inhibited the expression of menin protein, while the silencing of the endogenous miR-24-1, through the transfection of a specific 2′-O-methyl-RNA miR-24-1 antisense, resulted in an increased expression of menin, suggesting the existence of a direct negative feedback loop between miR-24-1 and menin, which could have a role in MEN1 tumorigenesis.

Later, in 2016, the same research group [29] demonstrated in BON1 cells that, in addition to the negative feedback loop between miR-24-1, *MEN1* mRNA, and menin, there was also a feedforward loop in which menin protein directly binds to the pri-miR-24-1 (the primary mRNA precursor of miR-24-1), promoting the DROSHA-mediated processing to pre-miR-24-1 and, thus, the biogenesis of mature miR-24-1. Moreover, the specific siRNA-induced silencing of menin expression in BON1 cells resulted in a complete suppression of pri-miR-24-1 expression, indicating an essential and direct role of menin in miR-24-1 synthesis, that is exerted by acting both at the transcriptional and at the post-transcriptional modification levels of the miR-24-1 synthesis. Conversely, when an overexpression of menin is induced in BON1 cells, this leads to an increased expression of mature miR-24-1. Authors showed that menin specifically binds to pri-miR-24-1, but not to pri-miR-24-2, and that the silencing of menin expression had no effect on the pri-miR-24-2 expression and the processing to pre-miR-24-2.

**Figure 1:**
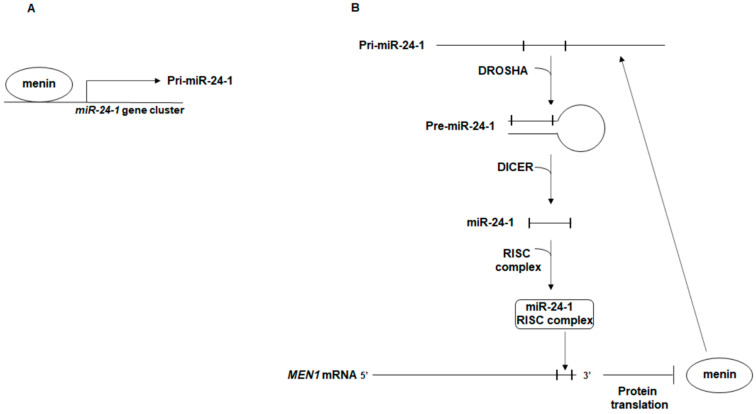
Schematic representation of the autoregulatory network between miR-24-1, *MEN1* mRNA, and menin. (**A**) menin binds the upstream region of the miR-24-1-encoding gene cluster on chromosome 9, promoting the transcription of pri-miR-24-1; (**B**) menin directly interacts with the primary transcript of miR-24-1, pri-miR-24-1, facilitating the DROSHA-mediated processing to pre-miR-24-1, in a positive feedforward loop in which menin promotes the maturation of the repressor of its own expression, miR-24-1. Mature miR-24-1, together with the RISC complex, binds the 3′ UTR of *MEN1* mRNA blocking translation of menin.

The proposed autoregulatory network between miR-24-1, *MEN1* mRNA, and menin is shown in Figure 1.

The regulatory network between miR-24, *MEN1*, and menin was demonstrated also in other species, than humans, in vitro and in vivo [30,31,32]. Interestingly, Gao et al. [30] demonstrated, both by Target Scan algorithm-based prediction and by an in vitro study in neonatal rat cardiac myocytes, that miR-24 targets the *CDNK1B* mRNA, blocking translation of the encoded p27^kip1^ protein, a negative regulator of cell cycle, resulting in a decreased G0/G1 arrest and in cell hypertrophy. Inactivating mutations of the *CDNK1B* tumor suppressor gene are responsible for the development of multiple endocrine neoplasia type 4 (MEN4), a clinical phenocopy of MEN1 [33]. Therefore, miR-24 could also have a role in MEN4 tumorigenesis.

### 2.1. miR-24, MEN1 mRNA, and Menin in Parathyroid Glands

Luzi et al. first demonstrated a direct regulation of menin expression by miR-24-1 in parathyroid tissues from MEN1 patients [27], suggesting an epigenetic oncogenic role of this miRNA in the tissue-specific tumorigenesis of these glands in *MEN1* mutation carriers. The analysis of miR-24-1 expression profiles in parathyroid adenomas from *MEN1* mutation carriers, in sporadic non-MEN1 tumor counterparts and in healthy parathyroid tissue, showed an inverse correlation in the expression profiles of miR-24-1 and menin, indicating a direct role of miR-24-1 in the post-transcriptional negative regulation of menin expression itself. This inverse correlation was present only when the wild type copy of the *MEN1* gene was still retained in the tumoral tissue, while in MEN1 adenomas presenting LOH at the 11q13 locus, menin resulted to be unexpressed and miR-24-1 was expressed at a very low level. The chromatin immunoprecipitation (ChIP) analysis, with an anti-menin antibody, in parathyroid tissues from MEN1 patients, showed the occupancy of miR-24-1 promoter region by menin, only in parathyroid adenomas conserving one wild type copy of the *MEN1* gene, but not in tumors presenting *MEN1* LOH, and it confirmed menin as a positive regulator of miR-24-1 expression, as previously demonstrated in BON1 cells [27].

Results from this study suggested that, in MEN1-associated parathyroid tumors, after the first inherited germinal “hit”, the somatic onset and progression of neoplasia could be under the control of a negative feedback loop between menin protein and miR-24-1. Authors hypothesized that this regulatory network could mimic and substitute the second somatic “hit” of tumor suppressor inactivation in tissues in which *MEN1* LOH has not yet occurred, probably representing an intermediate step before the irreversible genetic *MEN1* LOH. The pathway leading to MEN1 tumor development and progression could be explained by the proposed negative feedback loop between menin and miR-24-1 acting as a “homeostatic regulatory network” that needs to be “broken” to induce the somatic LOH “hit” and, consequently, the progression to neoplasia. This mechanism could explain the proliferative changes in the neuroendocrine cells (hyperplasia) that precede neoplasia, and this could also be hypothesized for pancreatic, duodenal, and other MEN1-associated tumors.

Some years later, the same Research Group [34] failed to find both miR-24-1 and miR-24-2 as differentially expressed between MEN1 parathyroid adenomas with somatic *MEN1* LOH at 11q13 and MEN1 parathyroid adenomas still retaining one wild type copy of the *MEN1* gene when they performed a microarray expression profiling covering about 1890 human miRNAs and using a *p*-value < 0.05 and a log_2_ fold change > 1.5 as parameters of statistical significance.

Grolmusz et al. [23] compared the expression of six potential *MEN1*-targeting miRNAs, including miR-24, in MEN1-associated parathyroid adenomas/hyperplasia with a germinal *MEN1* mutation (16), in sporadic counterparts bearing a somatic *MEN1* mutation (10) and in sporadic parathyroid lesions wild type for the *MEN1* gene (40). Expression levels of miR-24 and miR-28 were found to be significantly higher in sporadic primary hyperparathyroidism (PHPT) tissues with respect to MEN1-associated adenomas/hyperplasia, both when all the sporadic samples were considered and when considering the two sporadic PHPT subgroups of samples positive for nuclear menin staining or of samples negative for nuclear menin staining separately. No significant expression differences were found between the *MEN1* mutated vs. the *MEN1* wild type nuclear menin-negative sporadic lesions. All these data seemed to identify the higher expression of miR-24 and miR-28 as two universal signatures of sporadic non-syndromic parathyroid hyperplasia/adenoma, independent of their somatic genetic profile.

### 2.2. miR-24, MEN1 mRNA, and Menin in the Endocrine Pancreas

In the duodenum and the pancreas, the *MEN1* gene-associated heterozygote germline mutation causes hyperplasia of insulin- gastrin-, somatostatin-, and glucagon-secreting cells, resulting in a subsequent multifocal development of tumors. The great majority of tumors analyzed showed allelic deletion/inactivation of the second copy of the *MEN1* gene, whereas the precursor lesions retained their *MEN1* heterozygosity [35]. Pancreatic endocrine tumors develop from beta islets, through a stage of islet hyperplasia in which the wild-type *MEN1* allele is still retained. LOH at *MEN1* locus has never been found in these enlarged islets and, therefore, these lesions are considered to be a precursor stage of the tumors. It is possible that these pancreatic lesions present the same mechanism evidenced in MEN1 parathyroid glands: The proposed negative feedback loop between miR-24, *MEN1* mRNA and menin.

In 2014, Vijayaraghavan et al. [36] confirmed the presence of a feedback loop between miR-24 and menin, similar to that identified in parathyroids, in cell lines of endocrine pancreas (the MIN6 cells derived from a mouse insulinoma and the Blox5 cells, an immortalized cell line produced from a purified population of human pancreas beta cells by infection with retroviral vectors). Authors confirmed the direct interaction of miR-24 with the 3′UTR of *MEN1* mRNA. Cell transfection with pre-miR-24 increased miR-24 levels and resulted in a reduction of *MEN1* and menin expression, of 35% and 23% in MIN6 cells and 61% and 38% in Blox5 cells, respectively. Conversely, cell transfection with an anti-miR-24 antisense decreased endogenous miR-24 levels, in association with an increased *MEN1* expression in MIN6 cells but not in Blox5 cells, and with no significant difference of menin expression in both MIN6 and Blox5 cells. At the same time, an induced overexpression of menin increased the expression of mature miR-24 both in MIN6 (2.5-fold increase) and Blox5 (0.6-fold increase) cells, confirming that miR-24 and menin reciprocally regulate their expression. The negative effect of menin depletion on miR-24 expression was also confirmed in vivo in a conditional knockout mouse model with a selective deletion of the *MEN1* gene in pancreas beta cells, in which reductions of about 60% and 90% of miR-24 levels were found in heterozygote and homozygote knocked-out animals, respectively [36].

In the same study, since in the pancreas menin is known to regulate gene expression in a transcription complex with mixed-lineage leukemia (MLL) factor, the Authors [36] hypothesized the possibility that the miR-24-1- and miR-24-2-encoding genes could be targets of menin/MLL complex. The ChiP analysis in MIN6 and Blox5 cells showed that both menin and MLL proteins were present in the region upstream of miR-24, both on chromosomes 9 and 19, confirming the importance of the menin/MLL complex in miR-24 expression in pancreas islets.

Finally, since menin is known to negatively regulate cell proliferation by transcriptionally regulating expression of two main cell cycle inhibitors, p27^kip1^ and p18^ink4c^, the Authors also investigated the impact of miR-24 expression on these two proteins. Cell transfection with pre-miR-24 induced a 42% reduction of both p27 mRNA and protein in MIN6 cells, and of 57% p27 mRNA, but not protein, in Blox5 cells, and a reduction of 48% of p18 mRNA, but not protein, in Blox5 cells. No effect on both expressions of p18 mRNA and protein was found in MIN6 cells. Inhibition of miR-24 expression by transfection with an anti-miR-24 antisense did not significantly affect p18 mRNA nor protein expression in MIN6 and Blox5 cells [36]. The fact that an increased expression of miR-24 reduced the expression of p27 and p18 mRNAs suggested this miRNA as negatively regulating the expression of these two cell cycle inhibitors in an indirect manner, via the suppression of menin translation. In this way, the increased expression of miR-24, and the subsequent silencing of menin, lead to a significantly increased cell proliferation in Blox5 cells, but not in insulinoma-derived MIN6 cells, in which cell growth is already at such a high level as to not be further influenced by menin, p27, and p18 inhibition. Considering this, it can be assumed that an increase in miR-24 expression could be responsible for enhanced proliferation of beta-cells and hyperplasia of pancreas islets in the first stage of MEN1 tumorigenesis.

Molecular effects of miR-24 in parathyroid glands and endocrine pancreas and possible roles in MEN1 tumorigenesis, reported in the currently available studies, are summarized in Table 1.

### 2.3. miR-24, MEN1 mRNA, and Menin in Non-MEN1 Tumors

Menin has been found to exert its tumor suppression activity by blocking cell hyperplasia in tissues other than those commonly affected by MEN1 syndrome, such as prostate, breast, lung, liver, and stomach [37].

Using the A549 cells (a continuous cell line derived from a human adenocarcinoma of the alveolar basal epithelium of the lungs) and the NCI-H446 cells (a cell line derived from a human small-cell lung tumor), Pan et al. [38] also confirmed the interaction of miR-24 with the 3′UTR of *MEN1* mRNA and the miR-24-mediated significant silencing of menin expression in lung cancer.

The induced overexpression of miR-24 in A549 cells inhibited menin expression and upregulated SMAD3 and cyclin D1, stimulating cell proliferation and, at the same time, increased expression of Bcl-2 and inhibited expression of Bax, resulting in the inhibition of cell apoptosis [38]. Conversely, the induced silencing of endogenous miR-24 increased menin expression and downregulated SMAD3 and cyclin D1, inhibiting cell growth, and, at the same time, inhibited Bcl-2 expression and promoted Bax expression, allowing cell apoptosis [38]. In addition, the induced overexpression of miR-24, in both A549 and NCI-H446 cell lines, upregulated SMAD3 and MMP2 and significantly enhanced cell migration and invasion, while downregulation of miR-24 decreased the expression of both SMAD3 and MMP2, preventing cell migration and invasion. Taken together, these data indicate miR-24 as an oncogenic effector in lung cancer cells, acting as promoter of both cell proliferation and metastatic spread.

In the same study, the analysis of surgical specimens from human lung cancer showed a higher expression of miR-24, and a mirrored lower expression of menin, in tumors with respect to the adjacent healthy tissues. miR-24 expression inversely correlated with tumor size and patient overall survival, while patients with a higher menin expression showed a longer survival rate [38].

Bronchopulmonary carcinoids of the neuroendocrine cells of the bronchial epithelium arise in approximately 5–8% of MEN1 patients [39]. Despite the different cytological origin with respect to lung cancers studied by Pan et al. [38], an involvement of the miR-24-*MEN1* mRNA-menin network in the tumorigenesis of these carcinoids in MEN1 syndrome cannot be excluded.

Although menin has been found to regulate cell proliferation in hepatocellular carcinoma [40], the exact role of menin in liver carcinogenesis has not yet been widely studied, and the liver is not a site of primary tumor development in MEN1 syndrome, but only metastases from pancreas and duodenal MEN1-related neuroendocrine tumors. Ehrlich et al. [37] studied the miR-24-*MEN1* mRNA-menin network in six human cell lines of intrahepatic and extrahepatic cholangiocarcinoma (CCA), a biliary epithelial adenocarcinoma whose cells adopt a neuroendocrine-like phenotype during their proliferation. Both *MEN1* mRNA and menin expression resulted significantly decreased in all six CCA-derived cell lines compared to a human immortalized, non-malignant, cholangiocyte cell line (H69). A further-induced silencing of menin in tumoral cells increased cell growth, while the induced menin overexpression reduced the cell proliferation rate together with a decreased cell migration and invasion.

All the six tumoral cell lines showed a basal overexpression of miR-24 with respect to the H69 control, as well as human CCA tumor samples compared with their matched healthy tissues. The induced knock-down of miR-24 was associated with an increase of menin expression, a decreased expression of pro-angiogenic and pro-proliferative factors, and a reduction of cell proliferation and migration.

## 3. Targeting miR-24: A Potential Therapeutic Tool for MEN1 Tumorigenesis

The miR-24-driven post-transcriptional inhibition of menin synthesis, in parathyroid cells and pancreas beta islets, could represent an intermediate epigenetic step guiding the MEN1 tumorigenesis. menin has been demonstrated to have a role in the maintenance of genome stability and DNA repair, protecting cells from DNA damage [41,42]. At the same time, the epigenetic silencing of wild type menin by miR-24 promotes cell proliferation and exposes cells to DNA damage, which could result in deletion at the 11q13 locus or mutation of the second copy of the *MEN1* gene, favoring the somatic *MEN1* LOH, which represents a common hallmark of MEN1 cancers in parathyroids and endocrine pancreas.

In light of this, it is conceivable that targeting and silencing the expression of miR-24 at this intermediate, still reversible, step, before the occurrence of the genetic, irreversible, *MEN1* LOH, could restore expression and activity of wild type menin and block neoplastic initiation and progression, representing a promising innovative tool for preventing tumor development and progression in MEN1 patients [43].

Silencing of a miRNA that has an oncogenic activity (oncomiR), such as miR-24 in MEN1, can be obtained by miRNA inhibition target therapies, that include antisense anti-miRNAs (synthetic anti-miRNA oligonucleotides (AMOs), modified synthetic AMOs, locked nucleic acids (LNA)-based AMOs, and antagomirs), small molecules miRNA inhibitors, miRNA sponges, and miRNA masks. These anti-oncomiR tools, and their characteristics, are depicted in Table 2.

Among the above mentioned oncomiR inhibitor target therapies, transfection of target cells with RNA antagomirs [45] appears to be a promising tool for miR-24 silencing in MEN1. Synthetic RNA antagomirs for the in vivo silencing of endogenous miRNAs were first created in 2005; intravenous injection of these molecules in mice has been shown to considerably reduce the expression of target miRNA, with a silencing effect lasting up to three weeks after systemic administration [50]. Currently, this technology appears to be a promising tool for the treatment of different types of human cancer and other diseases [51,52,53], also thanks to the fact that antagomirs do not activate any immune response.

RNA antagomirs consist in single strand RNAs, complementary to the target miRNAs to be inhibited, presenting specific chemical modifications (introduction of a 2′-O-methyl residue to nucleotides and substitution of an oxygen atom with an atom of sulfur within the phosphodiester bonds; phosphorothioate bond), to reduce RNA degradation by endogenous ribonucleases and conjugated with a cholesterol tail at the 3′-end to enhance permeation of cell membrane and intracellular distribution [54,55].

To further improve the efficacy of this exogenous delivery system, it is possible to conjugate, through electrostatic interactions, the anionic RNA antagomir with polymeric cations possessing extremely low toxicity and immunogenicity (i.e., polyethylenimine; PEI), able to grant high efficiency of cell transfection, increase the bond to the target miRNA, and, at the same time, protect the transfected molecule from intracellular lysosomal degradation [56,57].

In addition, it is possible to conjugate the RNA antagomir-PEI complex with paramagnetic nanoparticles (PMNP), which give the RNA antagomir-PEI-PMNP complex the potential to be specifically conveyed in vivo, after a systemic intravenous injection, to target cells and tissues, in which we want to silence the target miRNA, through the application of external magnetic fields [58,59,60].

Luzi et al. [29] tested a 2′-O-methyl-modified antisense RNA complementary to hsa-miR-24-1 in the BON1 cells, measuring the effect of transfection of this molecule on both miR-24-1 and menin expression. Transfection of this antisense RNA caused a specific inhibition of activity of the endogenous miR-24-1, with a subsequent over-expression of menin, with respect to BON1 cells transfected with a scrambled sequence RNA as control.

These preliminary and promising results, obtained in basic research, show the ability of the anti-miR-24-1 antagomir to target and inhibit the human endogenous miR-24-1 and restore the normal expression of wild type menin, and prompt the possibility to develop and design RNA antagomir-PEI-PMNP complexes against hsa-miR-24, for systemic administration to MEN1 patients and prevention of tumor development.

A first attempt to evaluate in vivo the therapeutic effect of the miR-24 silencing on tumor growth was performed by Ehrlich et al. [37] in an athymic nude mouse model in which the CCA were induced by injection of Mz-ChA-1, a human cell line of extrahepatic CCA. After the development of tumors, treated mice were injected, three times a week, with a commercial miR-24 hairpin inhibitor, while the control group received an injection of a scramble (inactive) hairpin inhibitor. Ten weeks after the first injection, treated animals showed a significant reduction in tumor size compared to untreated controls, in association with a decreased nuclear Ki-67 staining in tumor specimens. Quantitative expression analysis, performed on tumor samples from treated and control mice, showed a decreased expression of both pro-angiogenic and pro-proliferative factors in treated animals. Results from this study clearly show that targeting miR-24 represents a promising therapeutic tool for reducing biliary tumor growth and suggest the possibility of using the same approach for other tumors, the tumorigenesis of which involves the oncomiR miR-24 and its inhibitory action on the *MEN1* tumor suppressor gene.

## 4. Future Research Needed in the Field of MEN1 Syndrome and miRNAs

In addition to miR-24, other miRNAs showed to be possibly involved in the MEN1 tumorigenesis or to be deregulated in the sporadic tumor counterparts of the neuroendocrine tissues commonly affected in MEN1 syndrome [25], and they should be further investigated by functional studies, as was done for miR-24, to assess their importance in MEN1-related tumor development and progression.

In this paragraph we briefly summarized the most promising miRNAs, that could be involved in the tumorigenesis of the three MEN1 main affected endocrine tissues.

Luzi et al. [34] profiled the expression of 1890 human miRNAs between MEN1 parathyroid adenomas with somatic *MEN1* LOH or still retain one wild type copy of the *MEN1* gene, and with respect to non-MEN1 sporadic parathyroid adenomas. After both the microarray analysis and the quantitative RT-PCR validation, three miRNAs were identified as differentially expressed between the two groups of MEN1 tumors. Authors analyzed, by ComiR tool, the putative gene targets of these three differentially expressed miRNAs, with a focus on genes known to be involved in parathyroid tumorigenesis. miR-4258 resulted remarkably downregulated in MEN1 parathyroid adenomas with *MEN1* LOH, with respect to those without *MEN1* LOH. miR-4258 was predicted to target the gene encoding the cyclin D1 (*CCND1*), a positive regulator of cell cycle and cell growth, suggesting that, in the absence of wild type menin, cells lose the miR-4258-driven negative control of *CCND1* expression, presumably leading to an uncontrolled cell growth. Conversely, miR-664 resulted to be upregulated in *MEN1* non-LOH adenomas and down-regulated in *MEN1* LOH adenomas, both with respect to non-MEN1 controls. miR-664 was predicted to target the *CDC73* tumor suppressor gene, whose germinal inactivating mutations are responsible for the hyperparathyroidism-jaw tumor syndrome [61] and somatic mutations have been associated with the development of sporadic parathyroid carcinoma [62], and the *CDKN2C* tumor suppressor gene encoding the p18^ink4c^, an inhibitor of the cyclin kinases CDK4 and CDK6, that negatively controls the cell cycle G1 progression. When a copy of the wild type *MEN1* gene is conserved, it can be speculated that the over-expression of miR-664 leads to silence these tumor suppressor genes, resulting in promoting cell growth and favoring tumor initiation. miR-1301 resulted upregulated in *MEN1* LOH parathyroid adenomas, both with respect to the *MEN1* non-LOH parathyroid adenomas and the non-MEN1 controls. miR-1301 was predicted to target the *CDKN1B* tumor suppressor gene, whose inactivating mutations are responsible for MEN4 syndrome, the *RET* oncogene, whose activating mutations are responsible for the multiple endocrine neoplasia type 2 (MEN2) syndrome, the *AP2S1* gene, whose loss-of-function mutations have been associated with familial hypocalciuric hypercalcemia type 3, the *CCND2* gene encoding cyclin D2 a positive regulator of cell cycle, and the *CTNNB1* gene encoding the catenin beta 1, involved in the regulation of cell adhesion. These data on expression and in silico analyses suggested these three miRNAs as potentially involved in MEN1 parathyroid tumorigenesis. The interaction of these three miRNAs with *MEN1* and menin protein should be functionally investigated, to better understand the exact role of these epigenetic regulators in the development and progression of parathyroid tumors, including their possible role in parathyroid carcinoma, which is extremely rare in MEN1 patients and appeared not to be directly correlated with the *MEN1* mutation. In addition, expression and functional role of these three miRNAs should be also studied in other MEN1 target tissues, to verify if their possible oncogenic role is tissue-specific only to parathyroid glands or, as for miR-24, they act in other MEN1-associated malignancies. Indeed, the *CDKN2C* gene, which could be a target of miR-664, has been demonstrated to be frequently lost in pituitary adenomas [63].

Regarding the endocrine pancreas, Lu et al. [64] showed that the miR-17, whose expression is induced by glucose and hyperglycemia, directly downregulated menin expression, through targeting the 3′UTR of the *MEN1* mRNA, promoting proliferation of the pancreas beta cells. On the other hand, *MEN1* is not only a direct target of miRNAs, but it has been showed also to regulate the biogenesis of some miRNAs that are involved in insulin-signaling and insulin-induced proliferation of pancreas beta cells. Through its direct physical interaction with the arsenite-resistance protein 2 (ARS2), a component of the nuclear RNA cap-binding complex that stabilizes pri-miRNA, menin facilitates the processing of pri-let-7a and pri-miR-155 to pre-let-7a and pre-miR-155 [65]. IRS2, a protein involved in the insulin-signaling pathway, is a target of mature let-7a, and resulted to be over-expressed when *MEN1* expression is lost. miR-155, whose biogenesis is positively regulated by menin, had been previously showed to be s among the top downregulated miRNAs in pancreatic neuroendocrine tumors [66], and its downregulation could have a role also in MEN1 tumorigenesis of the endocrine pancreas.

Two miRNAs, miR-15a and miR-16-1, that had been previously shown as having a significantly reduced expression in human sporadic pituitary adenomas [67], were reported to be downregulated also in MEN1 pituitary tumors from Men1^+/−^ mice, with respect to the healthy pituitary tissue [68]. The expression of these two miRNAs inversely correlated with the expression of cyclin D1 [68], suggesting their possible tumor suppressor gene-like role in pituitary tumorigenesis. The *Men1* knockdown in the AtT20 mouse pituitary cell lines resulted in a significantly decreased expression of miR-15a [68], suggesting that the decrease of miR-15a expression in the pituitary cells of MEN1 patients may be a direct result of menin expression lost, that may concur to MEN1 pituitary tumorigenesis. Study on human specimens and functional studies are needed to confirm these preliminary findings.

## 5. Conclusions

- The absence of a genotype-phenotype correlation in MEN1 syndrome suggested a possible role of epigenetic factors in the development of the individual clinical phenotype in any single patient, even in presence of the same *MEN1* mutation.
- miRNAs have shown increasing evidence of a direct role in human malignancies, both for sporadic and hereditary cancers. Several miRNAs resulted to be deregulated in the sporadic tumor counterparts of the neuroendocrine tissues commonly affected in MEN1 syndrome. A role of specific mi-RNA deregulation also in MEN1 tumorigenesis can be suspected.
- miR-24 has been demonstrated to negatively regulate menin expression in the parathyroids and the endocrine pancreas in MEN1 syndrome, and in other non-MEN1 sporadic tumors, suggesting it as initiator of menin loss-derived tumorigenesis.
- Targeting/silencing miR-24, during the hyperplastic phase of parathyroid and endocrine pancreas tumorigenesis and before the occurrence of the somatic *MEN1* LOH, could by a promising tissue-specific RNA-based anti-cancer therapy, aimed to restore the correct expression of menin in pre-cancerous cells.

## Figures and Tables

**Table 1:** Molecular effects of miR-24 parathyroid glands and endocrine pancreas, and possible roles in MEN1 tumorigenesis.

miR-24 Target	Effect	Possible Role in MEN1 Tumorigenesis	Reference
	**Parathyroid glands**		
*MEN1*	No effect on *MEN1* mRNA expression. Loss of menin protein expression.	Uncontrolled cell proliferation	[27]
	**Endocrine pancreas**		
*MEN1*	Reduction of both *MEN1* mRNA and menin expression.	Uncontrolled cell proliferation	[36]
*CDKN1B*	Reduction of expression of both *CDKN1B* mRNA and of p27^kip1^ protein in a mouse insulinoma cell line (MIN6).	Enhanced proliferation of beta-cells and hyperplasia of pancreas islets	[36]
*CDKN1B*	Reduction of expression of *CDKN1B* mRNA in an immortalized human pancreas beta cell line (Blox5). No data on expression of p27^kip1^ protein.	Enhanced proliferation of beta-cells and hyperplasia of pancreas islets	[36]
*CDKN2C*	No effect on expression of both *CDKN2C* mRNA and of p18^Ink4c^ protein in a mouse insulinoma cell line (MIN6).	Non applicable	[36]
*CDKN2C*	Reduction of expression of *CDKN2C* mRNA in an immortalized human pancreas beta cell line (Blox5). No data on expression of p18^Ink4c^ protein.	Enhanced proliferation of beta-cells and hyperplasia of pancreas islets	[36]

**Table 2:** miRNA inhibition target therapies for oncomiRs.

Therapeutic Tool	Description	Mechanism of Action on the Target oncomiR	Reference
Anti-miRNA oligonucleotides (AMOs)	Synthetic single-stranded RNA molecules complementary to the target miRNA	Competitive inhibition of the target mature miRNA by base pair	[44]
Modified AMOs	AMOs with a chemical modification of the 2′-OH into 2′-O′methyl- or 2′-O′methoxyethyl- groups, to increase intracellular stability	Competitive inhibition of the target mature miRNA by base pair	[44]
Antagomirs	AMOs with the 2′-O chemical modification and phosphorothioate bonds to increase intracellular stability and with a conjugated cholesterol tail at the 3′-end to favor cell membrane permeation	Competitive inhibition of the target mature miRNA by base pair	[45]
Locked nucleic acids (LNA)-based AMOs	AMOs containing an additional methylene link between the 2′-O atom and the 4′-C atom, that locks ribose into a more thermodynamically stable conformation	High-affinity base pair with their target mature miRNA. Inhibition of miRNA activity	[46]
Small molecules miRNA inhibitors	Small molecules (chemical compounds) that interfere with miRNA biogenesis and/or activity	Inhibition of a specific miRNA biogenesis and/or activity by chemical structure-based docking to miRNA precursor or to mature miRNA	[47]
miRNA sponges	RNA transcripts presenting multiple tandem repeats of the binding site (seed sequence) of the miRNA to be targeted	They stably interact with the endogenous target miRNA, preventing its interaction with its target mRNAs	[48]
miRNA masks	Single-stranded 2′-O′methyl-modified antisense oligonucleotides totally complementary to the miRNA binding sites in the 3′-UTR of the target mRNA	They “mask” the target mRNA from the endogenous miRNA, preventing the miRNA-driven suppression of protein translation	[49]
